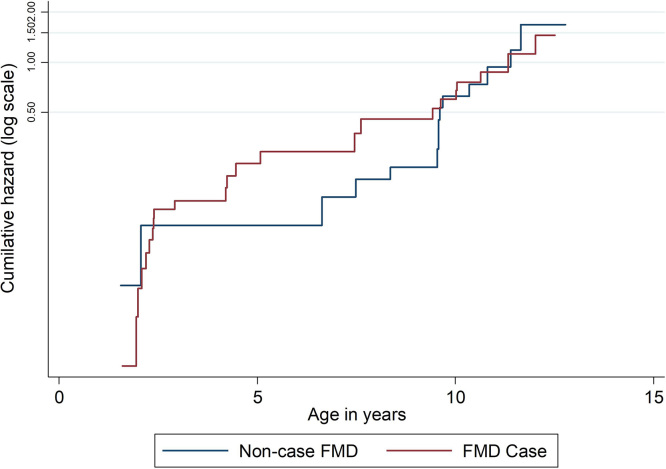# Impact of foot-and-mouth disease on fertility performance in a large dairy herd in Kenya

**DOI:** 10.1016/j.prevetmed.2018.08.006

**Published:** 2018-11-01

**Authors:** Gemma Chaters, Jonathan Rushton, Thomas Daido Dulu, Nicholas Anthony Lyons

**Affiliations:** a Veterinary Epidemiology, Economics and Public Health Group, The Royal Veterinary College, Hawkshead Lane, North Mymms, Hatfield, Hertfordshire AL9 7TA, UK; b State Department of Livestock, Ministry of Agriculture, Livestock and Fisheries, P.O. Private Bag Kabete, Kangemi 00625, Nairobi, Kenya; c The Pirbright Institute, Ash Road, Pirbright, Woking GU24 0NF, UK; d European Commission for the Control of Foot-and-Mouth Disease (EuFMD), Food and Agriculture Organisation of the United Nations, Viale delle Terme di Caracalla, 00153 Rome, Italy

**Keywords:** Foot-and-mouth disease, Economic impact, Fertility, Endemic

## Abstract

This was a retrospective cohort study using data collected from a large-scale dairy herd in Kenya (n = 328 female animals), to investigate the effects of foot-and-mouth disease (FMD) on herd fertility performance following a confirmed outbreak in a regularly vaccinated herd. Kaplan-Meier graphs were used to depict differences in survival functions between exposure groups and Cox regression models were used to calculate hazard ratios (HR) for associations between being clinical FMD cases and the following fertility outcomes: age at first calving; fertility failure related culling (not in calf); time to first service; time to conception. Potential confounding variables investigated and controlled for were age, breed, parity, stage of lactation/gestation and eligibility for service. A case control study was nested within the cohort to investigate the effects of disease on conception HR following calving by comparing animals susceptible to fertility suppression at the time of the outbreak (cases) to animals that had conceived prior to the outbreak (controls).

The median age of first calving in clinically affected young-stock was 2.7 months higher than non-clinical cases (adjusted HR = 0.37, 95%CI 0.21–0.67, P = 0.01). There was no evidence of a difference in fertility related culling and times to first service and conception. Animals susceptible to fertility suppression at the time of the outbreak had a lower hazard of conception compared to animals served prior to the outbreak (HR = 0.56, 95%CI 0.41–0.75, P = 0.01). Within the herd, the odds of being a case decreased with parity and age likely related to the lifetime number of vaccination doses received which may reduce the impact among older animals in the herd. Moreover, one would expect the impact to be higher in a non-vaccinating herd to be higher. Notwithstanding these limitations, the results of this study provide evidence that FMD outbreaks in endemic settings impact herd fertility performance. An increased age at first calving is likely to increase rearing costs and reduce an animal’s lifetime productivity while poorer conception rates will likely extend calving intervals. Impaired herd fertility and production will incur higher costs to the farmer and society as animals are less productive which for FMD can extend beyond the outbreak period where economic studies tend to focus. These impacts of FMD on herd fertility should be considered when conducting benefit-cost analyses of FMD control to inform resource allocation.

## 1 Introduction

Foot-and-mouth disease (FMD) is an economically devastating livestock disease putting major constraints on productivity and negatively impacting livelihoods across the world. Consequently, the OIE and FAO launched the Global FMD control strategy in 2012 (James and Rushton, 2002; FAO and OIE, 2012; Onono et al., 2013).

FMD is caused by an aphthovirus, from the picornaviridae family and there are seven immunologically distinct serotypes with limited or cross protection (Grubman and Baxt, 2004). The FMD virus is highly transmissible affecting cloven hoofed animals, including domestic cattle, sheep, goats and pigs in addition to many wildlife species (Kitching, 2002). Painful ulcerative lesions develop on mucosal surfaces (commonly on the dental pad and tongue) and on sparsely haired areas including the teats. Clinical disease typically presents as pyrexia followed by hyper-salivation, lip smacking, inappetence and lameness whilst in young animals acute gastroenteritis and myocarditis can occur and precede death before lesions are observed (Jones et al., 1997; Kitching, 2002; Grubman and Baxt, 2004).

Seventy percent of the world’s extreme poor depend on income from livestock farming and there are 162 million impoverished livestock keepers in Sub-Saharan Africa making up a large heterogeneous subset of the expanding global population (Thornton et al., 2003). Across all levels of the global livestock industry farmers should be producing on the slope of the marginal input-output production curve to achieve an optimum level of production. This is increasingly important as more demands are placed upon farming systems. Disease in livestock wastes energy that could otherwise be used for growth, reproduction and production. Disease inherently creates risk and uncertainty, leading to underinvestment and inefficiency in a system which is shown by a downwards shift of the production function curve (Ferranti, 2016).

The Global FMD burden mirrors the distribution of impoverished livestock keepers being endemic throughout many low and middle income countries that lack resources and infrastructure to eliminate it (Paton et al., 2009; Knight-Jones et al., 2016). In sub-Saharan Africa, estimates of FMD seroprevalence in cattle include 23% at the animal level and 54% at herd level in Ethiopia (Bayissa et al., 2011) and 52.5% in Kenya (Kibore et al., 2013). Losses incurred by disease can be classed as direct (both visible and invisible) and indirect through additional costs, revenue forgone and externalities (Rushton, 2011). There is a paucity of evidence in the scientific literature on the full socioeconomic impact of FMD as current knowledge relies on estimates from small studies in low and middle income countries focusing on direct losses such as milk production, change in weight gain and loss of draught power. Consequently, estimated losses of US$6.5 to 21 billion anually in endemic countries are likely to be an underestimate (Knight-Jones et al., 2016). Further comprehensive follow up studies are necessary to understand and quantify the true impact of the disease including the indirect costs and invisible loss burdens FMD places on individuals and societies.

The impact of FMD on fertility performance is categorized as an invisible loss as the effects are difficult to measure, particularly in the less intensive farming systems where FMD is endemic (Knight-Jones et al., 2016). Poor fertility performance of cattle leads to inefficiency in the farming system as more input per unit output is required. This combined with changes in herd structure results in less animal derived protein and micronutrients available for the individuals and societies depending on these nutritional sources (Knight-Jones and Rushton, 2013). If FMD affects fertility performance it could incur far greater costs to the industry and livelihoods than previously estimated. It is important that appropriate data are collected on the effects of FMD on fertility so that a robust economic analysis of FMD impact can be performed enabling policy makers to make informed decisions on resource allocation to mitigate disease impact (Knight-Jones and Rushton, 2013). The aim of this study was to utilise data from a large-scale dairy farm in Kenya, following an outbreak of FMD, to assess the impact on fertility performance using a rigorous and repeatable epidemiological approach. The objectives of the analysis was to use these data to explore the hollowing hypotheses:

• Clinical FMD in early life will increase the age of first calving
• Clinical FMD results in an increased risk of culling due to fertility failure
• Clinical FMD results in prolonged times to first service and conception following an outbreak
• FMD affects an animals time to conception if viral challenge occurs when a cow is in the pre-antral and antral stages of follicle development (i.e. susceptible to fertility suppression)

## 2 Materials and methods

### 2.1 Farm description

The study was conducted on a 240-hectare dairy farm located in Nakuru County, Kenya. This farm was selected due to the presence of detailed records on fertility management for individual animals before, during and after an FMD outbreak including clinical cases. At the time of the FMD outbreak there were 333 cattle on the farm: 4 adult bulls kept separate from the rest of the herd; one 18-month old bull kept with the dry cows; and 328 lactating cows, dry cows and heifers. Seventy-two percent of the herd was purebred Jersey and the remaining were Holstein-Friesian or crossbreds. Calves born on the farm remained with the dam for 12–24 h before removal to converted horse stables for two weeks until strong enough to go outside. Bull calves were euthanaised shortly after birth. The herd is primarily pasture-fed with cows milked twice daily through a milking parlour. At the time of the outbreak there were 171 (milking and dry) cows in the herd averaging 11 litres per cow per day and 157 calves and heifers.

Each animal kept on the farm had an individual record card corresponding with its ear tag number which manually recorded all individual health events, treatments and services. Farm policy was to first serve heifers at 15–16 months with an aim to have first calving as close to 24 months as possible. The voluntary waiting period after calving was 45 days before first service. Although the farm was aiming for a 365 day calving interval, there was no stringent cut off used for the number of serves an animal would have before being culled from the herd for poor fertility performance. Fertility data including dates and details of calving events, heat events and service events were copied from the record cards into a spreadsheet by the primary author (GC) and any discrepancies or missing data were looked up in the service diary and milking record sheets to maximise dataset accuracy. Pregnancy diagnosis was done by the farmer after no return to oestrus with the results of manual palpation and ultrasound recorded on each cows unique individual record card.

### 2.2 FMD management and outbreak

FMD is endemic in Nakuru County and frequently reported. On the study farm, cattle were routinely vaccinated against FMD with a locally produced, aqueous based, quadrivalent vaccine (Fotivax^™^, Kenya Veterinary Vaccines Production Institute, Embakasi) for serotypes endemic in the region (O, A, SAT 1, SAT 2). All cattle were included in the vaccination from the age of six months old and repeated every 4 months with no additional doses given to youngstock. Vaccination occurred 99 days prior to the index case for the outbreak considered in this study.

**Fig. 1:**
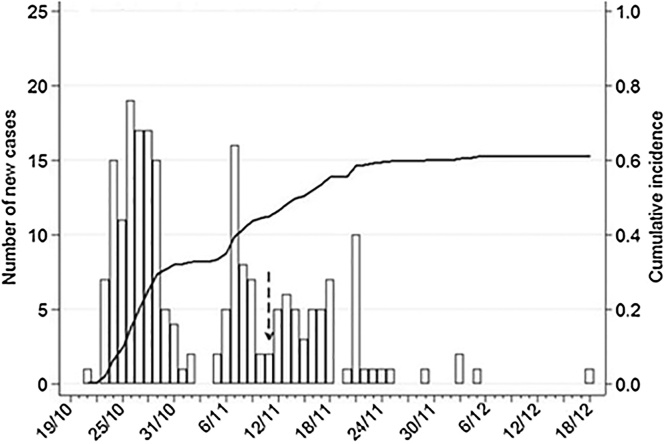
Epidemic curve and cumulative incidence of FMD cases. Denominator for cumulative incidence is the mean number of cattle present on the farm during the outbreak (21/10/13–18/12/13) and the dashed arrow indicates the date a reactive vaccine was given to all animals on the farm.

The index case of the FMD outbreak occurred on the 21/10/2013 and the last case recorded on the 18/12/2013, the date of onset of clinical signs for each cow was recorded by the herd manager (Fig. 1). Epithelium samples were sent to the National reference laboratory in Embakasi and FMDV serotype O was detected by antigen ELISA. The herd manager used the following case definition to record clinical cases: any animal hyper-salivating and showing a depressed appetite. Other clinical signs noted by the herd manager included ulcerative vesicular lesions in and around the oral cavity, teats and feet. All animals matching the case definition were treated according to the clinical signs. Animals with oral lesions were given oral *acetylsalicylic acid* and open foot lesions were sprayed with *oxytetracycline.* Vaccination was given to all cattle on day 24 of the outbreak as part of the four monthly schedule using the regular product. Initially clinical cases were moved to an isolation paddock but as the outbreak progressed cases were left in their management groups due to both lack of space and continued ease of management of the herd.

### 2.3 Study design

A retrospective cohort study approach was used to investigate associations between clinical FMD and fertility performance. The study start date was the date of the index case (21/10/2013) and 32 months of follow-up data were used up to the 21/06/2016. Cattle included in the study were all females present on the farm during the outbreak including those born. The primary exposure variable investigated for all outcomes was whether or not a cow was diagnosed by the herd manager with FMD during the outbreak period.

Outcomes investigated and associated inclusion criteria were:

• Age at first calving. All animals present on the farm that had not calved at the time of the outbreak were included. Study entry date is the date of the index case in FMD outbreak
• Culling due to fertility failure. Animals that were culled because they were not back in calf were classified as fertility failures. All females present on the farm during the outbreak were included. Study entry date is the date of the index case in FMD outbreak.
• Time to first service and time to conception. Only animals that were eligible for service at the time of the foot and mouth outbreak were included. Study entry date was the date of calving prior to the index case of the FMD outbreak.

The exit date for these outcomes was the date of the event being investigated. Animals were censored if they left the herd due to sale or death prior to any event being recorded.

Time to conception was further investigated by conducting a nested case control study to look for evidence of an association between timing of disease in relation to the stage of the reproductive cycle at the time of the outbreak and the conception hazard ratio (HR). The exposed group (cases) comprised cows eligible for service during the FMD outbreak. The unexposed groups (controls) were cows confirmed in-calf before the start of the outbreak. Only cows that conceive were included in the exposed and unexposed groups. Survival analysis was used to investigate the effects of the timing of FMD challenge on conception HR. Study entry was calving date prior to FMD outbreak and exit was the date of subsequent conception.

Time to event analysis using date of birth as the origin for all models accounted for age differences in the cohort throughout the study. Age which is an important potential confounder when investigating fertility performance (French et al., 2001). Other potential confounding variables considered were parity, breed, stage of lactation/gestation and eligibility for service (Table 1). Eligibility for service was divided into two groups: “Not eligible for service” included heifers under 420 days old and gestating animals at the start of the outbreak. “Eligible for service” were heifers aged over 420 days old and non-gestating cows at the start of the outbreak. Time of lactation/gestation affected was divided into 5 groups (Table 1) to account for the different stages of the fertility cycle, gestation and follicle development at the start of the outbreak. Vaccination status was not considered in the analysis because the farm used regular whole herd vaccination on a fixed date with consequent collinearity between age and the lifetime number of vaccine doses received.

**Table 1:** Univariable analysis of potential risk factors associated with being an FMD case. OR = Odds ratio. CI = Confidence Interval.

Variable	Category	Total Number	Number of cases	OR	95% CI	P-value
Breed	Holstein-Friesian	76	43	Reference	–	–
	Jersey	228	147	1.47	0.86–2.49	0.16
	Crossbred	28	16	1.02	0.43–2.46	0.96
Parity	0	157	121	Reference	–	–
	1	46	34	0.84	0.40–1.80	0.66
	2	26	10	0.19	0.08–0.44	0.00
	3	17	9	0.33	0.12–0.93	0.04
	>4	82	32	0.19	0.12–0.34	0.00
Stage of gestation or lactation at the time of outbreak	Non-served heifers	114	86	Reference	–	–
	Not in calf and <45 days calved	18	9	0.33	0.12–0.90	0.03
	Not in calf and >45 days calved	50	27	0.38	0.19–0.77	0.01
	0-200 days in calf	105	64	0.51	0.28–0.91	0.02
	>200 days in calf	41	20	0.31	0.15–0.65	0.00
Eligibility for service at time of outbreak^a^	Eligible for service	143	87	Reference	–	–
	Not eligible for service	185	119	0.86		0.52

a “Not eligible for service” included heifers under 420 days old and gestating animals at the start of the outbreak. “Eligible for service” were heifers aged over 420 days old and non-gestating cows at the start of the outbreak.

### 2.4 Statistical analysis

Survival analysis was performed using STATA 14.0 (StataCorp, Texas, USA). Unconditional odds ratios were computed to assess the distribution of cases among the primary exposure variable (FMD status) and each of the confounding variable sub groups. Kaplan-Meier survival curves were created to visualize survival functions to account for unsuccessful services and right censoring which can be high in dairy herds due to inherently high replacement rates. Univariable models were initially assessed using the Wilcoxon Rank test to look for evidence of a significant difference in the survival function of the comparative groups. The Wilcoxon test was used as earlier differences in the outcomes under investigation were expected to be greater and likely to move towards 1 as follow up time increased. This produces a more conservative probability result compared to other tests available as it gives more weight to the earlier observations.

Cox proportional hazards regression models were developed to quantify the impact of each of the exposure variables on the daily hazard of each of the three outcomes of interest (age at first calving, the number of days from the start of the outbreak to the date of being culled for infertility, and the number of days from calving to conception). Hazard ratios were computed allowing us to estimate the effect of each level of each exposure variable on the daily probability (‘hazard’) of each outcome event. Cox proportional hazards regression models allow one to estimate the effect of a given exposure on the daily hazard of outcome event, controlling for the effect of confounding variables that have been included in the model.

Variables associated with the exposure and outcomes in the univariable analysis were taken forward into multivariable models using a forward stepwise approach and likelihood ratio (LR) tests. Confounding was judged based on changes in the HR when the variables were included in the model. Adherence to the proportional hazards assumption (PHA) was assessed in all models using a combination of Nelson-Aalen plots and Schoenfeld residual tests (Stare et al., 2005).

## 3 Results

There were 211 cases of FMD among the 346 cows that were present in the herd at the start of the outbreak on 21 October 2013. The incidence of FMD over the 8-week outbreak period was 61 (95% CI 56 to 66) cases per 100 cows at risk. No data were available for two young animals present during the outbreak so they were excluded from the study.

There were 328 cattle at risk of fertility related culling at the beginning of the study and 37 fertility failure related cull events during the 511 cattle-years follow up time. There were 161 animals at risk of first calving at the beginning of the study with a total of 1760 cattle-months follow up time and 78 first calving events recorded during the follow up time. Results from the univariable analysis assessing potential risk factors associated with being a case of FMD are shown in Table 1. The odds of being a case of FMD was higher among lower parities and non-served heifers compared to all other ‘stage of lactation/gestation’ groups. There was no evidence that breed and “eligibility for service” was associated with being a case of FMD during the outbreak.

### 3.1 Age at first calving

**Fig. 2:**
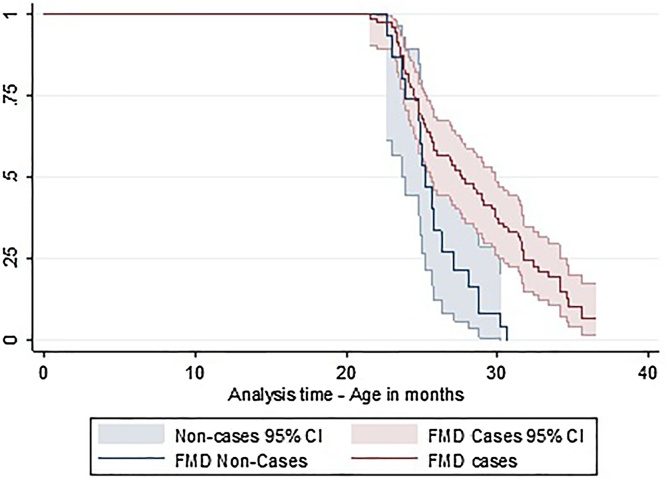
Unadjusted Kaplan-Meier survival curve for animals age at first calving. Animals were included in the study if they had not calved at the time of the FMD outbreak. 161 animals were included, 14 were not alive at the time of the FMD outbreak, so are included in the non-cases group. There were 78 1^st^ calving events during the total 1760 month follow up period. Animals were censored if they left the herd due to sale or death. The Y axis represents an animal’s cumulative probability of not having calved. Wilcoxon test for equity of survival function is p = 0.02. study origin was animals date of birth and date of entry to the study was the date of the index case (21/10/13).

Kaplan-Meier plots showed a difference in the age at first calving for FMD cases and non-cases (Fig. 2) and the Wilcoxon rank test revealed strong evidence of a difference between the two groups (median calving age: FMD cases = 27.7 months, Non-cases = 25.0 months, P = 0.02). A hazard ratio (HR) of less than one indicates that those in the exposed group had a higher age of first calving. Based on the univariable Cox regression analysis, FMD cases had 0.41 (95%CI 0.2–0.7, P = 0.01) the hazard of first calving (i.e. they calved older than the non-cases). Crossbred cows had a higher hazard of first calving (HR = 2.2, 95%CI 1.0–4.6) indicating that they calved earlier, compared with Holstein-Friesian (the baseline) and Jersey cattle (0.71, 95%CI 0.39–1.3). Tests for the proportional hazard assumption indicated that this was not violated in the models.

Multivariable analysis results for age at first calving are presented in Table 2. Breed was included in the model as it improved the fit based on a likelihood ratio test (P = 0.002). The results indicated that cases of FMD gave birth significantly older than non-cases indicated by an adjusted hazard ratio of 0.37 (0.21–0.67, P = 0.001). Comparing this to the crude HR 0.41, the confounding effects of breed were minimal. Based on Schoenfeld residuals there was no evidence that the proportional hazards assumption was violated (Table 2).

**Table 2:** Final multivariable Cox regression model for age at first calving in heifers that were recorded as being FMD cases compared to non-cases (n = 161). Total follow up time was 1760 months starting for each animal from the date of birth. Total number of calving events was 78.

Variable	Category	HR	SE	P-value	95% CI	Schoenfeld residuals P-value
FMD	Non-case	Reference	–	–	–	–
	Clinical case	0.37	0.11	0.001	0.21–0.67	0.37
Breed	Holstein Friesian	Reference				–
	Jersey	0.83	0.26	0.56	0.45–1.54	0.95
	Crossbred	2.81	1.09	0.01	1.31–6.01	0.35
Model Wald-P value = 0.01.						
Global Schoenfeld residuals P-value = 0.76.						

HR=Hazard ratio. SE = Standard Error. CI = Confidence Interval. Wald P-values are presented.

### 3.2 Fertility related culling

**Fig. 3:**
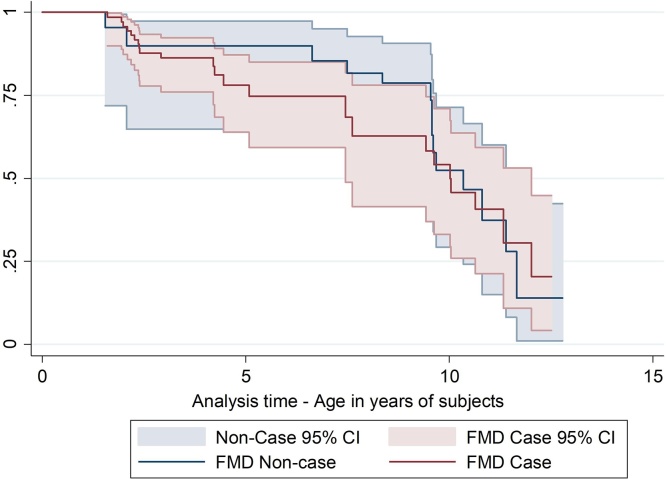
Unadjusted Kaplan-Meier survival curve for animals that exit the herd due to ‘fertility failure’ Animals included in the study were all animals present at the start of the FMD outbreak on 21/10/13 and all those born in the following 60 days. Number of subjects = 325, total follow up time is 510 years, fertility failures n = 37. Youngest age at entry to study time is 0 years and last age at exit is 12.7 years. Subjects were censored from the denominator if they left the herd during the study due to sale or death. Y axis is cumulative survival probability for an animal to have not left the herd due to being a fertility failure. Subjects origin and entry to the study date is ‘date of birth’.

Unadjusted Kaplan-Meier plots revealed no evidence of a difference in overall fertility related culling between cases and non-cases based on overlapping confidence intervals (Fig. 3) and a non-significant Wilcoxan rank test (P = 0.50). Visualization of the Nelson-Aelen plot (Supplementary material A) to evaluate the proportional hazards assumption indicated the lines crossed in younger animals (<2 years) and again in older animals (>10 years) where there are fewer observations, but are parallel in the midsection (for animals aged 2–10 years). Therefore only data from animals in this age period where the model assumptions are valid were considered in the analysis. In this age period, 10% of subjects in each group experienced fertility failure events (Supplementary material B).

The results of the univariable Cox regression analysis associating the effect of FMD and other variables on the hazard of fertility related culling is shown in Table 3. No evidence of a significant difference between the FMD cases and non-cases was detected. The proportional hazards assumption was met for all variables except breed that was not taken forward to the multivariable model. Stage of lactation or gestation at the time of the outbreak had a significant association with subsequent fertility failure related culling. Compared to the baseline group (non-gestating heifers) there was good evidence to suggest that gestating animals 1–200 days in calf at the time of the outbreak had the lowest hazard ratio for subsequently being culled due to fertility failure. In the early lactation, non-gestating groups there was no evidence of a difference in the hazard ratios compared to the baseline non-gestating heifer group. There was no evidence of a significant association between parity and fertility related failure events although this should also be interpreted with caution as age is already adjusted for using the Cox regression model. There was no evidence that eligibility for service status affected the hazard for culling due to fertility failure following the FMD outbreak.

**Table 3:** Results of univariable analysis using Cox proportional hazard regression models to explore the effects of FMD disease on the primary outcome “leaving the herd due to Fertility Failure” and the effects of other potential confounding variables in animals aged 2–10 years. HR = Hazard Ratio, SE = standard error, CI = Confidence Interval.

Variable	Category	HR	SE	P-value^b^	95% CI	P-value^c^	Schoenfeld residuals	Wilcoxon rank test
FMD	Non-Case	Reference	–	–	–	0.30	0.31	0.2
	Case	1.60	0.73	0.31	0.65–3.9			
Breed	Holstein-Friesian	Reference	–	–	–	0.25	0.03	0.07
	Jersey	2.35	1.76	0.256	0.54–10.2			
	Crossbred	0.79	0.97	0.85	0.07–8.8			
Parity	0	Reference	–	–	–	0.32	0.99	0.57
	1	1.20	1.21	0.86	0.17–8.7			
	2	1.03	1.46	0.98	0.06–16.6			
	3	0.01	–	–	–			
	>4	0.01	–	–	–			
Stage of gestation or lactation at time of outbreak	Not in calf heifers	Reference	–	–	–	0.01	0.85	0.04
	Not in calf and <45 days calved	0.41	0.47	0.44	0.04–4.0			
	Not in calf and >45 days calved	0.23	0.25	0.18	0.03–2.0			
	0-200 days in calf	0.12	0.12	0.05	0.01–0.96			
	>200 days in calf^a^	0.01	–	–	–			
Eligibility for service at time of FMD outbreak	Eligible for service	Reference	–	–	–	0.3	0.42	0.18
	Not eligible for service	1.6	0.66	0.3	0.68–3.6			

a No events in the >200 days in calf group so the hazard was less than 0.01 for these animals.

b Wald P-value for coefficient.

c likelihood ratio test.

The final multivariable Cox regression model results are shown in Table 4. There was no statistical evidence that FMD cases had a higher hazard ratio of culling due to fertility failure adjusted for the stage of lactation/gestation (HR = 1.7 (95%CI 0.66–4.2, P = 0.28) and there was no evidence of interaction between FMD and timing of infection, in relation to gestation/lactation stage (Likelihood Ratio (LR) test P-value = 0.31).

**Table 4:** Final multivariable Cox proportional hazards regression model showing the Fertility Failure rate of FMD cases and non-cases adjusted for stage of lactation/gestation, for animals aged 2–10 years present on the farm during the FMD outbreak. Model only includes animals aged 2–10 years in the risk set as animals outside this age range caused violation of the proportional hazards assumption. N = 248, cattle-years of follow up = 4165.7, number of Fertility failure events = 24. HR=Hazard ratio, SE = Standard Error, CI = Confidence interval. Wald P-values are presented.

Variable	Category	HR	SE	P-value	95% CI	Individual variant Schoenfeld residuals P value
FMD	Non-Case	Reference	–	–	–	
	Case	1.7	0.78	0.28	0.66–4.2	0.20
Stage of gestation or lactation at time of outbreak	Not in calf heifers	Reference	–	–	–	
	Not in calf and <45 days calved	0.40	0.47	0.43	0.04–4.0	0.89
	Not in calf and >45 days calved	0.24	0.26	0.19	0.03–2.1	0.93
	0-200 days in calf	0.11	0.12	0.04	0.01–0.93	0.57
	>200 days in calf	0.01^a^	–	–	–	–
Model Wald P-Value = 0.003						
Likelihood ratio test to include Time of infection P-value = 0.002						
Global Schoelfield Residuals Test P-value = 0.67						

a No events in the >200 days in calf group so the hazard was less than 0.01 for these animals.

### 3.3 Time to conception in the lactation following FMD outbreak

Univariable Cox regression of the conception hazard ratio (CHR) in animals that were not 0–200 days in calf during the FMD outbreak showed no evidence of an association with clinical FMD, breed or parity (Table 5). A hazard ratio of less than one indicates a variable was associated with delayed conception. Breed violated the proportional hazard assumption so was not considered further in the analysis. There was some evidence that lactation stage influenced CHR as animals eligible for service during the outbreak (over 45 days calved) had delayed time to conception HR = 0.47 (CI 0.23–0.96 p = 0.04). Lactation/gestation stage was added to the multivariable model to investigate the effects of FMD case diagnosis on CHR and it improved the model fit to the data (LR test P-value = 0.01) but there remained no evidence to support a difference in CHR between FMD cases and non-cases. In the multivariable model, adjusted conception hazard ratio in FMD cases was 0.86 (CI 0.50–1.5 P = 0.5)

**Table 5:** Cox regression Univariate analysis models for conception hazard ratio in all animals that were eligible for service on the farm during the FMD outbreak.

Variable	Category	HR	SE	P value^b^	95% CI	P- value^c^	Wilcoxon rank test P value	Schoenfeld residuals P Value
FMD	Non-Case	Reference				0.82	0.75	0.74
	FMD Case	0.94	0.25	0.87	0.56–1.6			
Stage of lactation or gestation at time of FMD outbreak	Not in calf and <45 days calved (n = 18)	Refernece	–	–	–	0.01	0.10	0.17
	Not in calf and >45 days calved (n = 50)^a^	0.47	0.17	0.04	0.23–0.96			
	>200 days in calf (n = 20)	0.98	0.36	0.96	0.48–2.00			
Parity	1	Reference	–	–	–	0.98	0.99	0.99
	2	1.0	0.53	0.97	0.36–2.8			
	3	1.0	0.74	0.99	0.23–4.3			
	4	1.3	1.2	0.79	0.23–6.7			
Breed	Holstein-Friesian	Reference	–	–	–	0.72	0.26	0.01
	Jersey	0.84	0.24	0.55	0.49–1.5			
	Crossbreed	1.2	0.68	0.78	0.38–3.7			

a Animals out of the voluntary wait period and thus eligible for service.

b Wald P-value for coefficient.

c likelihood ratio test.

### 3.4 Time to first service in the lactation following FMD outbreak

The hazard ratio for time to first service event was investigated for animals that had calved prior to the FMD outbreak and were classed as eligible for service during the FMD outbreak. Univariable Cox regression analysis results investigating the association between clinical FMD and potential confounding variables with the hazard of first service showed no evidence of any associations (Supplementary material C). None of the univariable models violated the proportional hazards assumption.

### 3.5 Conception animals exposed to virus challenge compared to pre-outbreak conditions

To investigate the effects of FMD on herd fertility performance further, a case-control study nested within the study cohort was performed to estimate the impact on conception rate. Cases were animals eligible for service at the time of the FMD outbreak and controls were animals that had calved prior to the outbreak and were already gestating during the outbreak. Analysis was done to look at the conception hazard ratio between these two groups to evaluate if the timing of potential virus challenge relative to the stage of the fertility cycle reduces the ability to conceive. Animals who were eligible for service at the time of the FMD outbreak had a CHR of HR 0.56 (95% CI 0.41–0.75) compared to animals that had conceived prior to the outbreak. FMD clinical diagnosis had no effect on the association, nor did breed or parity (Supplementary material D). Further multivariable analysis, adding parity and breed did not improve model fit when added in the forward stepwise direction,

## 4 Discussion

There is a paucity of literature on the impact of FMD on cattle fertility although this is an essential parameter for quantifying the long-term impacts of disease. Fertility is complex, influenced by many environmental and management factors meaning many parameters must be measured before, during and after an outbreak to build a picture of overall disease impact. The benefits of conducting a cohort study were that numerous fertility outcomes could be assessed simultaneously and exposure status was measured before the outcome, which eliminates recall bias and reverse causality.

Survival analysis of cohort data gives reliable and repeatable results that can be used to complement economic analysis for policy and decision-making. (Lee et al., 1989; Lean et al., 2016). These methods are also able to adjust for the effects of age by using date of birth as the origin. It was considered essential that age be included in the models *a priori* due to the strong association between age and the number of vaccinations received by individuals that may lead to increasing level of immunity acquired with age.

In heifers, the median age of first calving was 2.7 months higher in FMD cases, supported by the adjusted HR for first calving in FMD cases of 0.37 (95%CI 0.21–0.67, P = 0.01) indicating that the instantaneous risk of calving during the study period was significantly lower than non-affected cattle. This may have resulted from reduced feed intake and growth rates related to clinical disease although inflammatory disease can cause endocrinopathies that result in a poor quality dominant follicle, corpus luteum and subsequent conception failure (Butler, 2003; Ferguson et al., 2012). The most efficient age at first calving for a dairy heifer for maximum lifetime productivity is 23–24 months (Pirlo et al., 2000; Nilforooshan and Edriss, 2004). Lower age at first calving reduces the cost of rearing replacements, increases the odds of survival to older parities and reduces the risk of culling, improving herd production efficiency (Tozer and Heinrichs, 2001; Berry and Cromie, 2009; Hultgren and Svensson, 2009). This studies result showing an increased age at first calving for FMD cases also supports findings from other studies showing that heifers that are ill during the rearing period calve later than those not ill during the rearing period (Waltner-Toews et al., 1986).

Comparing FMD cases and non-cases did not reveal any difference in the time to first service and conception although the overall number of subjects included in this part of the analysis was reduced to 109 and the total number of cases in this group was 56 resulting in more unstable models and higher standard errors. This analysis was extended to a nested case-control approach to further investigate the potential wider impact of the outbreak on the time to conception (for example due to subclinical infection in non-cases or due to changes in management during the outbreak period). For cows that were eligible for service at the time of the FMD outbreak the daily hazard of conception was 0.56 (95% CI 0.41–0.75) times that of the daily hazard of conception for cows submitted for service during the pre-outbreak period. This could be due to increased inflammatory mediators suppressing fertility, or because of pyrexia and mouth lesions reducing feed intake and adding to postpartum nutritional imbalances, further impairing fertility. More work needs to be done to investigate the effects of subclinical disease and herd level challenge on subsequent fertility performance but this result indicates that viral challenge in a herd reduces a cows ability to conceive if eligible for service during the outbreak. Delayed time to conception increases the herd calving interval above the optimal 12–13 months which reduces the herds overall productivity, as more input per unit output is required (Schmidt, 1989; Garnsworthy, 2004). In addition to the direct effects on herd productivity, increased calving interval reduces the number young animals produced per year which could have further negative effect on the wider community who purchase surplus young stock from the herd.

There was no evidence for an association between being recorded as a clinical case of FMD and culling due to fertility failure. The analysis was limited by the low number of events (n = 24) used in the final analysis resulting in a large standard error and unstable model. A previous study in Kenya found weak evidence of an association between clinical FMD and subsequent culling one year later (Lyons et al., 2015). This potential impact of FMD requires further exploration using data from more farms to better quantify this effect on longevity and productivity.

There are numerous limitations to this study that should be considered when making inferences from the results. These include: a) a high number of censored observations during the study period with the assumption that they left the study for reasons not related to the primary outcome; b) the study was performed on a herd that used regular vaccination so the estimated effects may be lower than an unvaccinated herd; c) this is a single farm outbreak so the results cannot be reliably generalized to make inferences about the effects of FMD on fertility performance in the wider cattle population and d) clinical cases relied upon herd manager recording and judgment so there is some potential for misclassification that may bias the results; e) farmers may be more inclined to retain less fertile cows in the herd due to other losses related to the FMD outbreak (for example chronic lameness or mastitis); f) cattle that did not conceive were not included in the nested case-control study which may underestimate the disease impact. The reasons for poor vaccine performance in this herd are unknown but may include such factors as low potency, poor match between the virus and field strain and poor maintenance of the cold chain (Lyons et al., 2015).

## 5 Conclusion

Fertility failure losses are estimated to cost a farmer 10% of his income (Dijkhuizen et al., 1985).

This unique study adds to that empirical evidence showing that FMD impairs fertility and livestock productivity by both increasing the age of first calving and increasing the time to conception in cows challenged by the disease when eligible for service. Both impacts may incur large costs to the farmer over the months and years following an outbreak not necessarily evident at the time. The true economic impacts of FMD could be far higher than previously estimated and economic analysis of FMD impacts should include the costs incurred due to reduced fertility performance. Additionally, farmers should be made aware of the full and extended consequences of FMD infection on their herd so they are able to make sound management decisions based on evidence.

This study supports the argument that more international support and collaboration is needed to push towards FMD elimination and eradication so that farmers, currently suppressed under the burden of FMD, can reduce their vulnerability, improve productivity, meet the growing nutritional demands of the expanding population and potentially gain access to international markets which could boost economies and alleviate poverty (Perry and Grace, 2009).

## Figures and Tables

**Figure:**