# Supplementary material for: Impact of foot-and-mouth disease on fertility performance in a large dairy herd in Kenya
Source: Prev Vet Med. 2018 Nov 1;159:57–64. doi: 10.1016/j.prevetmed.2018.08.006 (PMC6193135; doi:10.1016/j.prevetmed.2018.08.006)
Supplement: Supplementary file 1 [file mmc1.docx]

**Supplementary material B.** Number of fertility failure (not getting in calf) cull events by FMD status and the age distribution, in years, of the subjects in each group at study entry and exit.

| Category | Number of subjects | Number of fertility failures^a^ | Years of follow up contributed | Earliest entry age | Mean entry age | Mean exit age | Last exit age |
| --- | --- | --- | --- | --- | --- | --- | --- |
| FMD Case  Age 2-10 years | 159 | 15 (10%) | 248 | 0.1 | 2.8 | 4.3 | 9.8 |
| Non-Case  Age 2-10 years | 89 | 9 (10%) | 168 | 0 | 4.4 | 6.2 | 9.9 |

^a^ Animal is culled from the herd due to not getting in calf. As the follow up time is reduced to only look at follow up experiences of animals aged between 2-10 years there are n=24 events in total between the two exposure groups.
