# Supplementary material for: Impact of foot-and-mouth disease on fertility performance in a large dairy herd in Kenya
Source: Prev Vet Med. 2018 Nov 1;159:57–64. doi: 10.1016/j.prevetmed.2018.08.006 (PMC6193135; doi:10.1016/j.prevetmed.2018.08.006)
Supplement: Supplementary file 2 [file mmc2.docx]

**Supplementary material C**. Univariate Cox regression model Wald P value, Schoenfeld residuals P value and Wilcoxon Rank sum test P values for all models investigating the Hazard ratio for first service. Animals were included if eligible for service at the time of the FMD outbreak and had calved at least once prior to the outbreak.

| **Exposure variable** | **Model Wald P-value** | **Wilcoxon Rank Sum P-value** | **Schoenfeld residuals P-value** |
| --- | --- | --- | --- |
| FMD | 0.66 | 0.87 | 0.72 |
| Stage of lactation/gestation at time of FMD outbreak | 0.35 | 0.37 | 0.99 |
| Parity | 0.63 | 0.29 | 0.72 |
| Breed | 0.40 | 0.64 | 0.52 |
