# Supplementary material for: Impact of foot-and-mouth disease on fertility performance in a large dairy herd in Kenya
Source: Prev Vet Med. 2018 Nov 1;159:57–64. doi: 10.1016/j.prevetmed.2018.08.006 (PMC6193135; doi:10.1016/j.prevetmed.2018.08.006)
Supplement: Supplementary file 3 [file mmc3.docx]

**Supplementary Material D.** Results of univariate cox regression survival analysis model on the conception hazard ratio using a case control nested within the study cohort. Animals were followed from their calving prior to the FMD outbreak to the time of conception or leaving the herd. A comparison was made between those eligible for service at the time of the FMD outbreak (cases) to those who had already conceived prior to the outbreak (controls). HR=Hazard ratio, CI=Confidence Interval.

| **Variable** | **Category** | **Conception HR (95% Cl)** | **Model Wald P Value** | **Schoenfeld residuals P-value** | **Wilcoxon Rank Sum P-value** |
| --- | --- | --- | --- | --- | --- |
| Eligibility for service | Not eligible for service during the outbreak | Baseline | 0.01 | 0.94 | 0.01 |
|  | Eligible for service during the outbreak | 0.56 (0.41-0.75) |  |  |  |
