# Supplementary material for: Impact of foot-and-mouth disease on fertility performance in a large dairy herd in Kenya
Source: Prev Vet Med. 2018 Nov 1;159:57–64. doi: 10.1016/j.prevetmed.2018.08.006 (PMC6193135; doi:10.1016/j.prevetmed.2018.08.006)
Supplement: Supplementary file 4 [file mmc4.docx]

**Supplementary material A.** Map of the study farm indicating paddock locations (grazing and crop growing paddocks), buildings (where the parlour is located) and the perimeter electric fence. An access road ran around the inside of the perimeter fence so animals in outer paddocks were at least 3m from the perimeter electric fence restricting contact with outside animals.

**Supplementary material A.** Nelson-Aalen plot showing the cumulative hazard (on the log scale) for exiting the herd due to ‘Fertility Failure reasons’ against ‘Age of animal in years’ for FMD cases and Non-cases to check the proportional hazards assumption.

**Supplementary material B.** Number of fertility failure (not getting in calf) cull events by FMD status and the age distribution, in years, of the subjects in each group at study entry and exit.

**Supplementary material C**. Univariate Cox regression model Wald P value, Schoenfeld residuals P value and Wilcoxon Rank sum test P values for all models investigating the Hazard ratio for first service. Animals were included if eligible for service at the time of the FMD outbreak and had calved at least once prior to the outbreak.

**Supplementary Material D.** Results of univariate cox regression survival analysis model on the conception hazard ratio using a case control nested within the study cohort. Animals were followed from their calving prior to the FMD outbreak to the time of conception or leaving the herd. A comparison was made between those eligible for service at the time of the FMD outbreak (cases) to those who had already conceived prior to the outbreak (controls). HR=Hazard ratio, CI=Confidence Interval.
